# Supplementary material for: Oxygen Saturation in Hospitalized COVID-19 Patients and Its Relation to Colchicine Treatment: A Retrospective Cohort Study with an Updated Systematic Review
Source: Medicina (Kaunas). 2023 May 12;59(5):934. doi: 10.3390/medicina59050934 (PMC10223566; doi:10.3390/medicina59050934)
Supplement: Supplementary file 1 [file medicina-59-00934-s001.zip › medicina-2365494-supplementary.pdf]

**Table S1.** Treatment for COVID-19 patients according to Egyptian protocol.				
protocol	Protocol September 2021		Protocol November 2020	
severity	Moderate	Severe	Moderate	Severe
Antivirals	Hydroxychloroquine 200mg<br>(400 mg twice on first day then 200 mg daily for 6 days<br>+<br>Ivermectin 6 mg<br>(36 mg on day 0-3-6)<br>OR<br>Favipravir 1600mg twice daily 1st day then 600 mg twice daily 5-10days<br>+<br>Ivermectin 6 mg<br>(36 mg on day 0-3-6)<br>OR<br>Remdesivir 100 mg<br>(200 mg on the first day,<br>then 100 mg daily for 9 days for high-risk patients	Remdesivir 100 mg<br>(200 mg on the first day, then 100 mg daily for 9 days)	Hydroxychloroquine 200mg, (400 mg twice on first day then 200 mg daily<br>+<br>Ivermectin 6 mg (36 mg on day 0-3-6)<br>OR<br>Lopinavir/Ritonavir (200/50 mg) (2 tablets bid)<br>OR<br>Remdesivir 100 mg<br>(200 mg on the first day,<br>then 100 mg daily) for high-risk population with SaO_2 < 92	Remdesivir 100 mg<br>(200 mg on the first day, then mg 100 mg daily<br>OR<br>Lopinavir/Ritonavir (200/50 mg) (2 tablets bid)
Supplements	-Zinc: 50 mg daily<br>-Vitamin C: 1 gm daily<br>-Lactoferrin: 100 mg twice daily<br>-Acetyl Cysteine: mg TDS200	-	-Zinc: 50 mg daily<br>-Vitamin C: 1 gm daily<br>-Lactoferrin: 100 mg twice daily<br>-Acetyl Cysteine: mg TDS 200	-Zinc: 50 mg daily<br>-Vitamin C: 1 gm daily<br>-Lactoferrin: 100 mg twice daily<br>-Acetyl Cysteine: mg TDS200
Anticoagulation	Prophylactic anticoagulation<br>Or<br>Therapeutic if D-dimer >1000 ng/mL	Prophylactic anticoagulation<br>OR<br>Therapeutic anticoagulation (Consider D-dimer level as a guide)	Prophylactic anticoagulation<br>If D-dimer between 500-1000 ng/mL<br>OR<br>Therapeutic anticoagulation<br>If D-dimer >1000 ng/mL	Prophylactic with anticoagulation<br>If D-dimer between 500-1000 ng/ mL<br>OR<br>Therapeutic with anticoagulation<br>If D-dimer > 1000 ng/ Ml or severe hypoxia
Anti-inflammatory	Steroids if patients are dyspneic or CT SCAN showed significant deterioration dexamethasone 6 mg or its oral equivalents.<br><br>Colchicine 500Mg/12 hours for 1month	Steroids<br>Methyl prednisolone 1mg/kg/ day<br><br>Tocilizumab 4-8 mg/kg/day<br>Max 2 doses<br>or<br>Sarilumab 200 mg single dose<br><br>Colchicine 500Mg/12 hours for 1month	Steroids<br>(if patient has severe dyspnea)<br>RR>24 or CT scan showing rapid deterioration.<br>Dexamethasone 6 mg or its oral equivalent	Steroids<br>(Dexamethasone 6 mg or methyl prednisolone (1 mg / kg /24 hours)<br><br>Tocilizumab4-8 mg/kg/day for 2 doses 12 to 24 hours apart after failure of steroid therapy to improve the case for 24 hours

Table S2. Database search strategy (March 2023):

Database	Keywords	Articles (Filter from 2020)
Scopus	(KEY ("Coronavirus") OR KEY ("Coronavirus infections") OR KEY ("COVID 2019") OR KEY ("SARS2") OR KEY ("SARS-CoV-2") OR KEY ("SARS-CoV-19") OR KEY ("severe acute respiratory syndrome coronavirus 2") OR KEY ("coronavirus infection") OR KEY ("severe acute respiratory pneumonia outbreak") OR KEY ("novel CoV") OR KEY ("2019 ncov") OR KEY ("sars cov2") OR KEY ("cov2") OR KEY ("ncov") OR KEY ("COVID-19") OR KEY ("COVID19") OR KEY ("coronaviridae") OR KEY ("coronavirus")) AND KEY (colchicine)	**246**
Research square	Colchicine restricts to covid-19	**73**
Clinical trial .gov	Covid, Colchicine	**36**
Research Gate	Colchicine AND covid*	**93**
PubMed	("Coronavirus") OR ("Coronavirus infections") OR ("SARS-CoV-2")) OR ("SARS-CoV-19")) OR ("COVID 2019")) OR ("SARS2")) OR ("severe acute respiratory syndrome 2" coronavirus)) OR ("Coronavirus infection")) OR (" severe acute respiratory pneumonia outbreak ")) OR (" novel CoV")) OR (" 2019 ncov")) OR (" sars cov2")) OR (" cov2")) OR (" ncov")) OR (" COVID 2019")) OR (" COVID-19")) OR (" coronaviridae")) AND (Colchicine)	**381**
Medrxiv	Coronavirus, SARS2, covid-19, colchicine	**128**

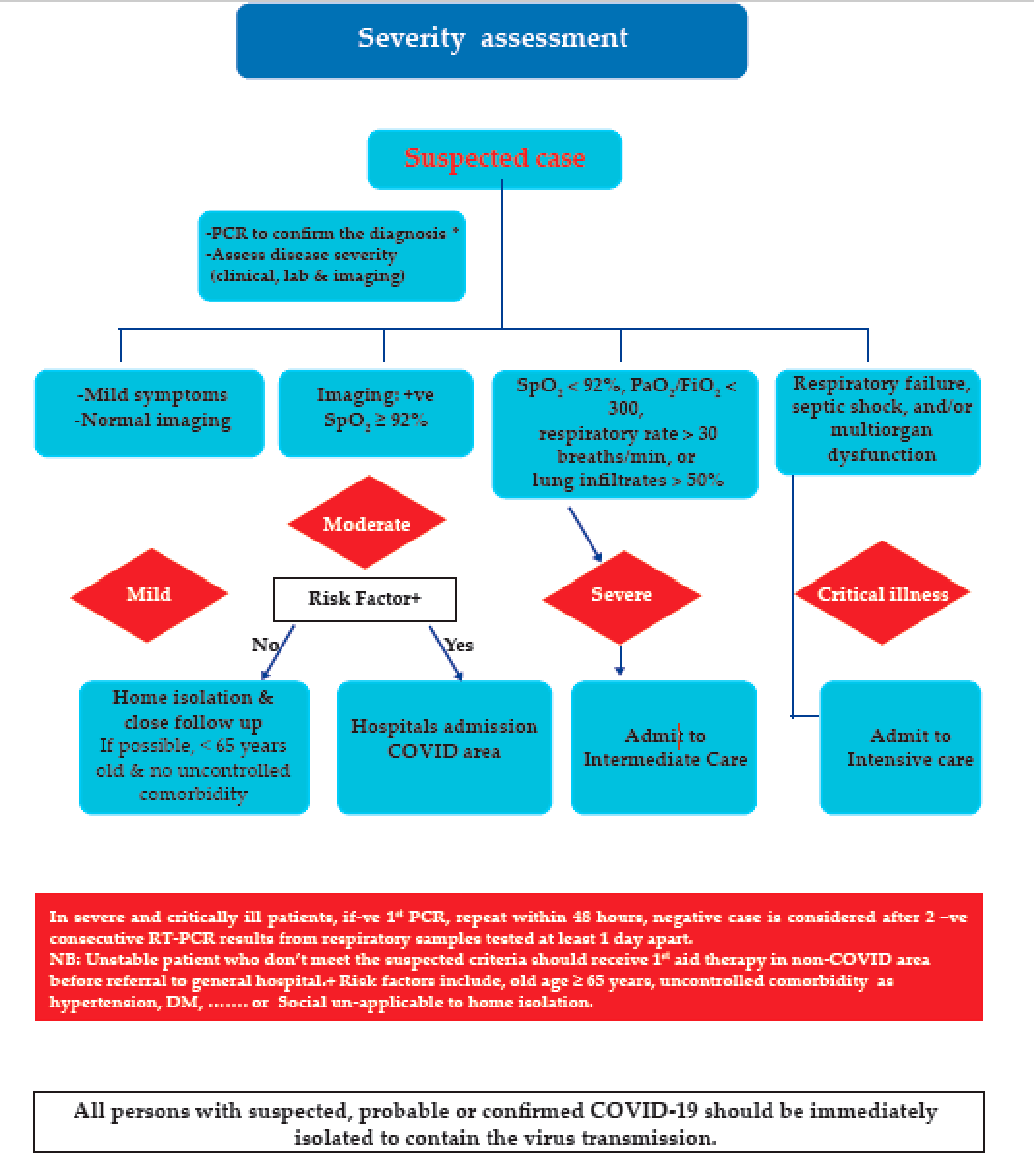

Figure S1. COVID-19 Severity assessment
